# Essential Oils Derived from *Momordica charantia* Seeds Exhibited Antiulcer Activity against Hydrogen Chloride/Ethanol and Indomethacin

**DOI:** 10.1155/2021/5525584

**Published:** 2021-04-22

**Authors:** Nurul 'Ain Abu Bakar, Muhammad Nazrul Hakim Abdullah, Vuanghao Lim, Yoke Keong Yong

**Affiliations:** ^1^Department of Biomedical Science, Faculty of Medicine and Health Sciences, Universiti Putra Malaysia, 43400 Serdang, Selangor, Malaysia; ^2^Integrative Medicine Cluster, Advanced Medical and Dental Institute, Universiti Sains Malaysia, Bertam, 13200 Kepala Batas, Penang, Malaysia; ^3^Department of Human Anatomy, Faculty of Medicine and Health Sciences, Universiti Putra Malaysia, 43400 Serdang, Selangor, Malaysia

## Abstract

*Momordica charantia* (MC) is popular for its medicinal uses especially for treating diabetic-related complications. However, the antiulcer activity of essential oil derived from the seeds has not been systematically studied. This study aims to evaluate the gastroprotective activities of essential oil derived from the seed of MC induced by hydrochloride acid/ethanol (HCl/EtOH) and indomethacin and pylorus-ligation model. Gastric ulceration was induced by oral administration of HCl/EtOH solution or indomethacin on day 7 after animals have been pretreated with testing compounds. The first group received just distilled water and the second group received ranitidine (100 mg/kg). Groups 3, 4, and 5 received 10, 50, and 100 mg/kg of essential oil based on their body weight (10 mL/kg), respectively. Macroscopically, pretreatment of essential oil extracted from MC significantly decreased ulceration induced by HCl/EtOH and indomethacin *in vivo*. Microscopically, essential oil also significantly suppressed the formation of edema, epithelial disruption, and mucosa erosions. Moreover, essential oil significantly elevated the pH without decreasing the total acidity of the gastric juice and was able to increase the amount of adherent mucus compared to control. Current results provide scientific basis to the ethno-pharmacological usage of the MC in preventing ulcer formation induced by HCl/EtOH and indomethacin.

## 1. Introduction

Gastric ulcer, one of the chronic diseases affecting millions of people worldwide, is erosion of the mucosal integrity of the stomach which extends through the muscularis mucosa into submucosa or deeper resulting from various factors including reductions in the blood flow. The condition imbalances between aggressive and defensive factors and increases release of reactive oxygen species in the gastric mucosa [1–4]. The current therapeutic drugs used to treat gastric ulcers are not fully effective and produce various adverse effects such as hypersensitivity, arrhythmia, impotence, and haemopoietin disorders [3, 5]. Furthermore, the increase in NSAIDs use in recent years could be the reason for the increase of gastric ulcer cases [6]. Thus, there is a need for more effective and safe anti-ulcer agents.

Diverse studies have shown that most of the herbal drugs will reduce the offensive factors and proved to be safe, clinically effective, better patient tolerance, relatively less expensive, and globally competitive [7]. There are many plants with ethno-pharmacological background that have been used in traditional medicine known to possess antiulcer properties, including *Phyllanthus niruri* [8] and *Euphorbia umbellata* [9].

*Momordica charantia* L., or commonly known as bitter melon or bitter gourd, belongs to the Cucurbitaceae family, and native to the semi-tropical climate of Thailand, India, and Africa. Apart from consuming it as vegetable, it has been traditionally used as folk remedy specially to treat diabetic-related complications. The whole plant of *M. charantia*, from the fruit to its roots, is known to have medicinal values. It has been used in treating eczema, gout, jaundice, psoriasis, pneumonia, and peptic ulcers, besides diabetes [10]. The biological activities of *M. charantia* (MC) were studied extensively, and interestingly, it is widely best known for the anti-diabetic activity [11]. Apart from that, it is also documented to possess antifungal [12], antiulcer [13], antioxidant effects [14], antihyperlipidemic [15], antimutagenicity [16], and antiviral [17] properties.

Based on the previous studies, *M. charantia* has been shown to have antiulcer activity [18, 19]. However, the evidence was reported from the fruit extract, but not on its seeds. Therefore, this present study is aimed at investigating the antiulcer properties of essential oil isolated from *Momordica charantia* L. seeds.

## 2. Materials and Methods

### 2.1. Chemicals and Reagents

Hydrochloric acid and indomethacin were purchased from Sigma, Malaysia, while the chemicals and reagents used for histology study such as DPX Mountant, haematoxylin and eosin, 37% formalin, 99.5% ethanol were from System, Malaysia; Alcian blue, diethyl ether, paraffin from Merck, Germany and xylene from BDH Chemicals, UK.

### 2.2. Preparation of Essential Oils

Essential oils extracted from the seeds of MC were a gift from Magna Bio-Laboratories Sdn. Bhd. Malaysia (Batches 37/11, 121/11, and 375/11). Extraction method from Noguchi et al. [20] and Kanan [21] was adapted.

### 2.3. Experimental Animals and Ethic Statement

A total of thirty-six male Sprague-Dawley rats, weighing 150 g–200 g obtained from the Animal House, Faculty of Medicine and Health Sciences, Universiti Putra Malaysia, were used in this study. The animals were housed in a group of six in the standard cages and maintained in a controlled environment (12:12-hour light and dark cycle) and temperature (25°C ± 3°C). The animals were fed with standard pellet diet and water *ad libitum* throughout the experiment. The experiments were performed in accordance with the guidelines and were approved by the Institutional Animal Care and Use Committee (IACUC), Faculty of Medicine and Health Sciences, Universiti Putra Malaysia (UPM/FPSK/PADS/BR-UUH/00275). All animals were acclimatized for at least one week before the experiments.

### 2.4. Antiulcerogenic Activities

#### 2.4.1. Hydrogen Chloride/Ethanol- (HCl/EtOH-) Induced Ulceration

The experiment was performed based on a slight modification of the method described by Mizui and Doteuchi [22]. Briefly, experimental animals were fasted for 24 hours with free access to water. Gastric ulceration was induced by oral administration of hydrogen chloride/ethanol (HCl/EtOH, 1 mL/60% EtOH containing 150 mM HCl) solution, 1 mL orally, for all the groups of animals on day 7 after being pretreated with testing sample for 1 hour. The animals were randomly divided into six experimental groups of six rats each. The first group received distilled water only without any inducement, the second group received distilled water but induced with HCl/EtOH (disease control), and the third group (standard) received reference drug, ranitidine (100 mg/kg). Groups 4, 5, and 6 received 10, 50, and 100 mg/kg of MC essential oils (MCEO) based on their body weight (10 mL/kg), respectively. All the treatments were given once daily for 7 consecutive days.

On the 7^th^ day, the rats were euthanized 3 hours after the induction of gastric ulcer under an overdose of ether and the stomachs were immediately excised. The stomachs were gently rinsed with normal saline to remove the gastric contents and blood clots. Then, the stomachs were dissected to examine macroscopically for haemorrhagic lesions. The number and severity of the ulcers were observed microscopically. Scoring system reported in Miñano et al. [23] was used to quantitate the ulcer index.

#### 2.4.2. Indomethacin-Induced Ulceration

The experiment was performed according to Jainu and Devi [24] with slight modification. Briefly, the groupings, duration of treatment, and dosage were the same as the aforementioned procedures. On the 7^th^ day, all the 24 hours' fasted rats were administered with 1 mL of indomethacin solution (100 mg/kg, dissolved in distilled water) 1 hour after pretreatment. After 6 hours, the rats were sacrificed and the stomachs were removed. Each stomach was examined macroscopically for ulcer lesion and the ulcer index of each animal was calculated based on the method mentioned above [23].

#### 2.4.3. Histopathological Examination of Gastric Tissues

Gastric tissues were fixed in 10% formalin solution for 24 hours. Sections of 5 *μ*m thickness were then stained with haematoxylin and eosin (H&E) followed by mounting with DPX mounting medium. Slide specimens were then observed under the light microscope for microscopic evaluation. The severity of ulceration was scored based on the scoring system reported by Farrell et al. [25].

#### 2.4.4. Pylorus Ligation-Induced Gastric Ulcer

In order to investigate the underlying mechanism of MCEO in treating pyloric ligation-induced gastric ulcers in rats, method from Shay et al. [26] was adapted. The ulcer formed in the gastric was scored, as in the previous studies [23], and the ulcer index for each animal was calculated as the mean ulcer score.

#### 2.4.5. Measurement of Gastric Acidity

Gastric content obtained from each pylorus-ligated animal was centrifuged for 10 min at 2500 rpm. A total of 1 mL of clear gastric content was then analysed for hydrogen ion concentration by titration with 0.01 N NaOH. Gastric acidity was expressed in mEq/L.

#### 2.4.6. Determination of Gastric Wall Mucus Content

Adherent gastric mucus was determined as described by Corne et al. [27] by adding Alcian blue solution to the gastric juice. The amount of Alcian blue was determined by using a spectrophotometer with OD 580 nm.

### 2.5. Statistical Analysis

Data were analysed using one-way analysis of variance (ANOVA). Mean comparison among the treatments and inducers were analysed using the Dunnett test at *p* < 0.05 considered as significant.

## 3. Results

### 3.1. Macroscopic Evaluation

#### 3.1.1. Effect of MCEO on HCl/EtOH-Induced Gastric Ulcer

**Figure 1:**
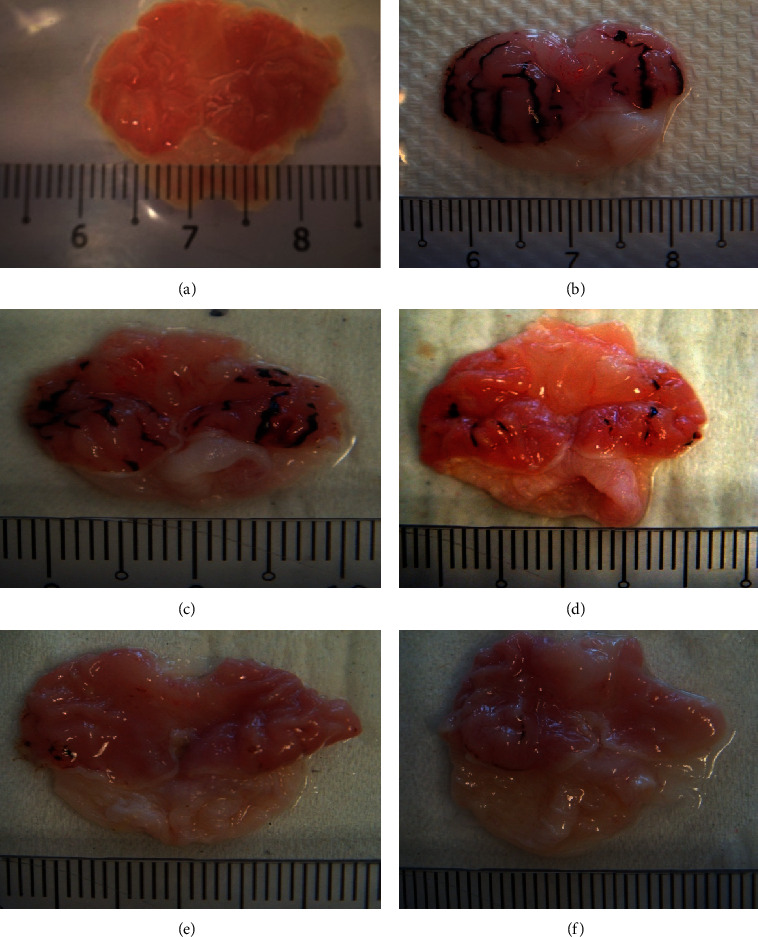
Gross appearance of the gastric mucosa in rats induced by HCl/EtOH. (a) Rats pretreated with distilled water only. (b) Rats with HCl/EtOH and distilled water. (c) Rats with HCl/EtOH and ranitidine. (d) Rats with HCl/EtOH and 10 mg/kg of MCEO. (e) Rats with HCl/EtOH and 50 mg/kg of MCEO. (f) Rats with HCl/EtOH and 100 mg/kg of MCEO.

HCl/EtOH at the dose of 1 mL has significantly induced ulcer formation in the disease control animals (Table 1 and Figure 1). Furthermore, the ulcer index in the disease control group was also significantly higher than the rest of the groups at 4.0 (Table 2). However, animals treated with MCEO at all concentrations significantly reduced the number of ulcers and ulcer index (Tables 1 and 2), showing a dose-dependent manner. Among all the concentrations, 100 mg/kg of MCEO possessed the highest activity, with 91% reduction on ulcer lesions compared with disease control group. Meanwhile, as for ulcer index, 41.75% inhibition was observed compared with the disease control (Table 2). Based on the gross appearance, gastric mucosa obtained from the treated group with 100 mg/kg of MCEO clearly showed protection by MCEO, with few ulcers observed (Figure 1). On the other hand, the standard group (ranitidine) also showed a significant reduction in ulcer lesion and index, however, much lesser than the overall concentrations of MCEO, with only 32% and 12.50% reductions in total area of lesion and ulcer index, respectively (Tables 1 and 2).

#### 3.1.2. Effect of MCEO on Indomethacin-Induced Gastric Ulcer

**Figure 2:**
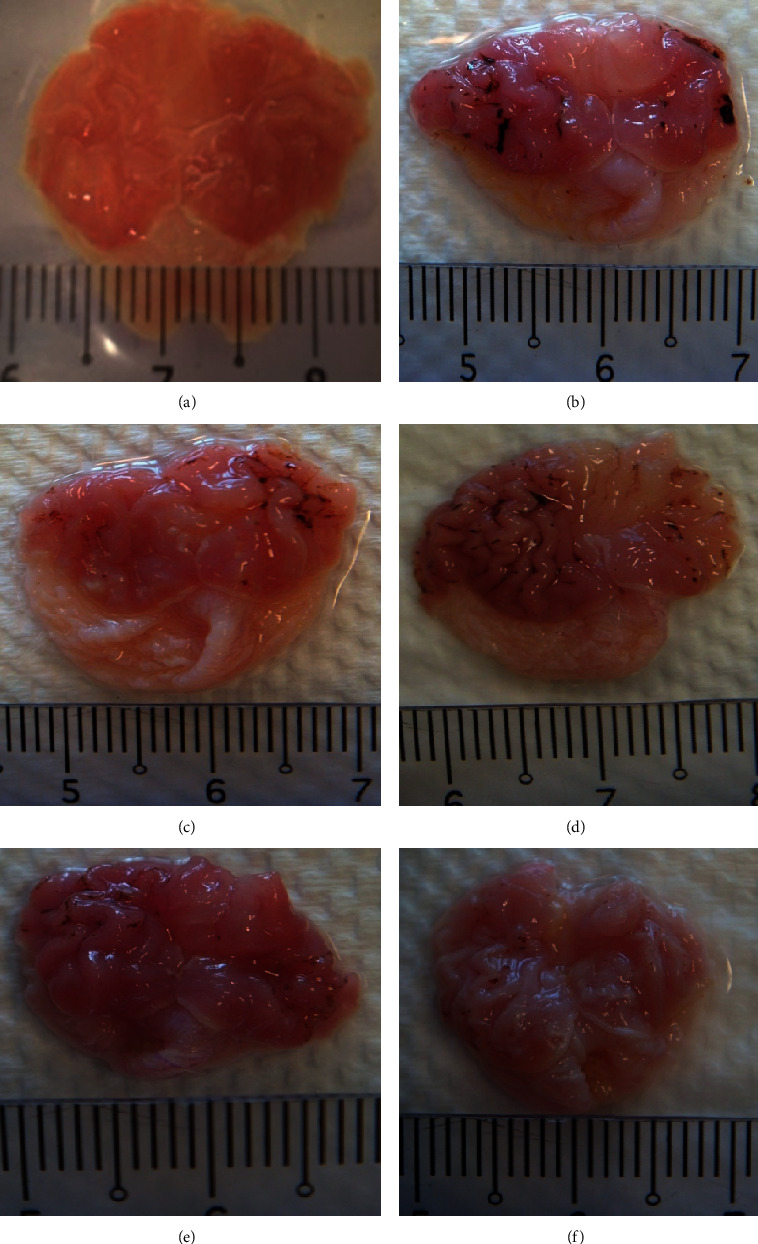
Gross appearance of the gastric mucosa in rats induced by 100 mg/kg of indomethacin. (a) Rats pretreated with distilled water only. (b) Rats with indomethacin and distilled water. (c) Rats with indomethacin and ranitidine. (d) Rats with indomethacin and 10 mg/kg of MCEO. (e) Rats with indomethacin and 50 mg/kg of MCEO. (f) Rats with indomethacin and 100 mg/kg of MCEO.

As shown in Table 3, MCEO significantly reduced the total lesion area induced by 100 mg/kg of indomethacin on rats in dose-dependent manner. Compared with all the treatments, 100 mg/kg of MCEO exhibited the highest percentage of anti-ulcerogenic effect (80.0%), comparable to the reference drug, 100 mg/kg of ranitidine (83.2%). From the gross appearance, indeed, all the concentrations of MCEO and ranitidine reduced the ulceration compared to the disease control group (Figure 2). On the other hand, based on the ulcer scoring system, only 100 mg/kg of MCEO and ranitidine showed significant difference compared with disease control group, while 10 and 50 mg/kg of MCEO did not show significant difference compared with disease control group, despite slight reduction in the ulcer index (Table 4).

### 3.2. Histological Evaluation of Gastric Lesions

#### 3.2.1. Effect of MCEO on HCl/EtOH-Induced Gastric Lesions

**Figure 3:**
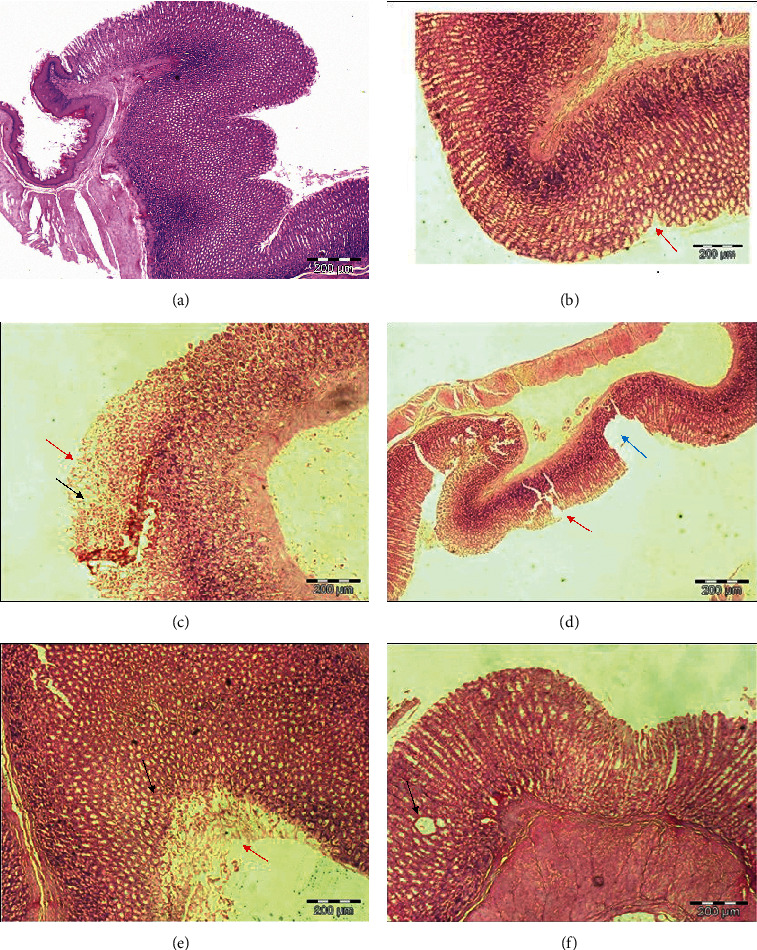
Microscopic evaluation of the gastric mucosa in rats induced by HCl/EtOH. (a) Rats pretreated with distilled water only. (b) Rats with HCl/EtOH and distilled water. (c) Rats with HCl/EtOH and ranitidine. (d) Rats with HCl/EtOH and 10 mg/kg of MCEO. (e) Rats with HCl/EtOH and 50 mg/kg of MCEO. (f) Rats with HCl/EtOH and 100 mg/kg of MCEO. The sections were cut parallel to the muscle layer. Red arrow indicates epithelial disruption; black arrow indicates oedema; and blue arrow indicates the erosion extending to the muscularised mucosae. H&E staining, 100x magnification.

Histological evaluation was performed to confirm the morphological changes in gastric tissues. As expected, there was extensive damage to the gastric mucosa showing prominent features of oedema and vacuolation, epithelial disruption, and mucosa erosions (Figure 3) in the disease control group. Based on the scoring system, the oedema or vacuolation, epithelial disruption and erosion scores in the disease control group were much higher than the treatment groups (Table 5). Surprisingly, the group treated with 100 mg/kg of MCEO significantly decreased the formation of oedema, epithelial disruption, and mucosa erosions (Figure 3 and Table 5). Also, the group treated with 100 mg/kg of ranitidine successfully reduced oedema and vacuolation formation compared with the disease control group (Table 5).

#### 3.2.2. Effect of MCEO on Indomethacin-Induced Gastric Lesions

**Figure 4:**
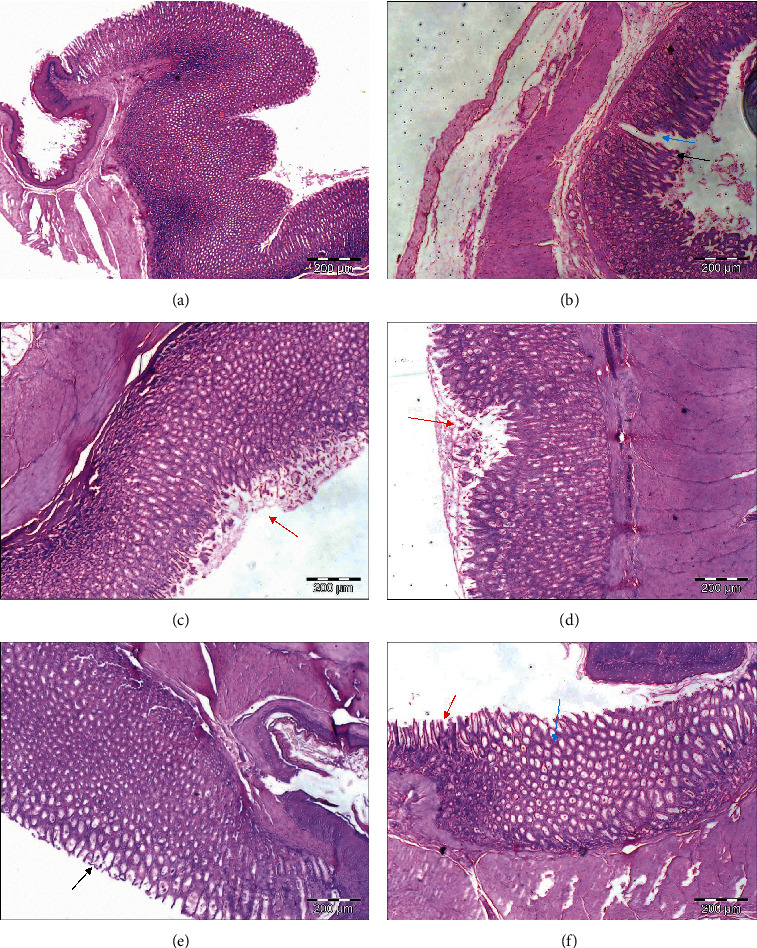
Microscopic evaluation of the gastric mucosa in rats induced by indomethacin. (a) Rats pretreated with distilled water only. (b) Rats with indomethacin and distilled water. (c) Rats with indomethacin and ranitidine. (d) Rats with indomethacin and 10 mg/kg of MCEO. (e) Rats with indomethacin and 50 mg/kg of MCEO. (f) Rats with indomethacin and 100 mg/kg of MCEO. The sections were cut parallel to the muscle layer. Red arrow indicates epithelial disruption; black arrow indicates oedema; and blue arrow indicates the erosion extending to the muscularised mucosae. H&E staining, 100x magnification.

Based on the histopathological analysis, the formation of oedema or vacuolation, epithelial disruption, and mucosae erosion were observed in the disease control group induced with indomethacin only (Table 6 and Figure 4). MCEO pretreated and reference drug groups showed better preservation of the gastric mucosa. Again, the highest concentration of MCEO (100 mg/kg) exhibited significant reduction in all aspects of ulceration (Table 6). MCEO seemed to arrest the ulcerative processes immediately after the mucosal layer was exposed to indomethacin. Furthermore, ranitidine group has also significantly increased the mean score for normal features while reducing the ulceration scores (Table 6).

### 3.3. Effect of MCEO in Pylorus Ligated-Induced Ulceration in Rats

In order to evaluate the antiulcer mechanism of actions, MCEO was examined by pyloric ligature-induced gastric ulcers in rat model using parameters including ulcer area, gastric volume, gastric pH, and total acidity (Table 7). Oral administration of MCEO and ranitidine at 100 mg/kg significantly reduced the total ulcer area (mm^2^) compared with disease control group (43% and 46% of protections, respectively). Nevertheless, all the concentrations of MCEO failed to increase the gastric volume. However, 50 and 100 mg/kg of MCEO significantly elevated the pH without decreasing the total acidity. Oral treatment of MCEO at concentrations of 50 and 100 mg/kg caused an increase in the amount of adherent mucus compared to the disease control group (Table 8). As expected, ranitidine enhanced the mucus production; however, its effect is much lesser than MCEO.

## 4. Discussion

Ethanol is one of the causative agents that lead to gastric ulcer formation and mucosa erosion [28]. In addition, studies have also reported that the administration with the combination of HCl/ethanol induced ulceration by increasing lipid peroxidation in mucosa layer with depletion of endogenous antioxidants [29]. It is believed that free radical's production is the aetiology of HCl/ethanol-induced gastric ulcer formation by altering gastric antioxidant defence system. Present data showed that the acidified ethanol led to gastric haemorrhagic erosion in rats. The total area of gastric lesions and ulcer index in the disease control group (treated with distilled water only) were significantly higher than the rest of the treatment groups. In microscopic evaluation, the acidified ethanol also caused significant vacuolation formation, epithelial disruption, and erosion, extending to muscularised layer. However, the gastroprotective efficacy of the MCEO (10, 50, and 100 mg/kg) was evident from significant reduction in the total area of gastric lesions. These data showed that the MCEO was effective in preventing acidified ethanol-induced gastric ulcer, which suggests its cytoprotective effect. Apart from that, it is also known that ethanol altered the gastric mucosa cellular integrity which is associated with oxidative stress and mitochondrial damage [30]; thus, based on the observations, MCEO was able to prevent the acidified ethanol-induced gastric ulcer by attenuating oxidative stress or antioxidant effect. Similar findings from the reported studies, showing essential oils, have been proven to prevent gastric mucosal ulceration in rats using this model [31, 32].

Over-the-counter non-steroidal anti-inflammatory drugs (NSAIDs) are used to relieve pain and inflammation. However, frequent and long-term administration of NSAIDs, such as aspirin and indomethacin, could become a risk factor of gastric ulceration. NSAIDs cause damage to the gastric mucosa via several mechanisms, including topical irritant effect on epithelium, disruption of mucosa barrier properties, inhibition of prostaglandin synthesis, decrease in mucosal blood flow, and interference with the re-epithelisation of the ulcer [33]. Previous studies reported that indomethacin reduced gastric mucosa protein, glutathione, catalase, and superoxide dismutase with an increase in lipid peroxidation in animals' studies [34]. Moreover, indomethacin-induced gastric mucosa damage could also be associated with reactive oxygen species [35]. Gastric prostaglandins play an important role in protecting the mucosal layers by stimulating the secretion of mucus, maintaining local blood flow, and increasing the resistance of epithelial cells to be damaged by cytotoxins [36]. However, suppression of prostaglandins by indomethacin increased the susceptibility to gastric mucosal lesions, as shown by disease control group in the current study. Interestingly, oral administration of MCEO in rats has significantly suppressed the damage to mucosa at all concentrations tested when compared with the disease control group. This demonstrates that MCEO is able to protect the gastric mucosa and suggests the possible involvement of prostaglandins and mucus productions. Sathishsekar et al. [11, 14] have reported that *M. charantia* seed extract significantly inhibited the toxic effects caused by free radicals by enhancing the enzymatic antioxidant defence system, such as glutathione (GSH), superoxide dismutases (SOD), and catalase (CAT). Thus, it can be postulated that the inhibitory properties of MCEO may also be due to its antioxidant activity and the ability to scavenge reactive oxygen species produced by indomethacin.

In order to determine the mechanism of actions, MCEO was tested on pylorus ligation-induced ulcer model. It was reported that the ulcer formation in gastric is due to the imbalance between offensive and defensive mucosal factors [37]. Therefore, it is an ideal model to examine the mechanism on how a drug works as an anti-ulcerogenic agent. The main cause is due to the accumulation of gastric acid and pepsin, which lead to the damage on the gastric mucosal barrier [38]. Based on the results, only 100 mg/kg of MCEO and ranitidine produced a significant reduction in the ulcer area with 43% and 46% of protections, compared to the disease control group. In addition, insignificant reduction in gastric volume was observed in all tested concentrations of the MCEO compared to the disease control group. However, significant increases were observed in pH, total acidity, and gastric wall mucus. Therefore, the protection offered by MCEO in the pylorus ligation setting may be due to acid neutralising effect and the upregulation of mucus secretion. These properties may be partly due to the fact that the phytochemical constituents present in MCEO, such as sesquiterpenes, phenylpropanoids, monoterpenes, and trans-nerolidol, were the major constituents found in the essential oil of *M. charantia* which were revealed by the previous studies [39]. Current data also showed that the reference drug, ranitidine, which was used for this study successfully suppressed ulcer induced by EtOH/HCl and indomethacin in all parameters. On top of that, effect of the highest concentration of MCEO (100 mg/kg) was shown to be comparable to ranitidine, suggesting that MCEO protects gastric ulcer formation which might be associated with inhibition of histamine H2 receptor. However, further experiments need to be carried out in order to confirm this hypothesis.

## 5. Conclusion

The results obtained from this study suggested that MCEO could evidently suppress the ulcerations induced by acidified ethanol, indomethacin-induced, and pylorus-ligated. Thus, this study has proved that MCEO is found to exhibit anti-ulcer potential via suppression of free radicals, upregulation of prostaglandins synthesis, and mucus secretion, which favours the healing process for gastric ulcers. These activities may be partly contributed by the phytochemical constituents, present in the MCEO. However, further research is needed for better understanding of the mechanisms and the active bio-compounds present in this essential oil in order to provide a new alternative for the clinical management of gastric ulcers.

## Figures and Tables

**Table 1:** Effects of MCEO on total lesion area (mm^2^) of gastric ulcer induced by HCl/EtOH in rats.

Treatments	Dose (mg/kg)	Total lesion area (mm^2^)	Inhibition (%)
Disease control (distilled water)	—	59.7 ± 7.4	—
Ranitidine	100	40.5 ± 3.2^*∗*^	32.1
MCEO	10	18.0 ± 4.0^∗∗∗^	69.8
	50	7.8 ± 3.4^∗∗∗^	86.9
	100	5.0 ± 1.9^∗∗∗^	91.6

Data are expressed as mean ± SEM, *n* = 6, ^*∗*^*p* < 0.05, and ^∗∗∗^*p* < 0.005. Dunnett's test as compared to disease control value.

**Table 2:** Effect of MCEO on ulcer index of gastric ulcer induced by HCl/EtOH in rats.

Treatments	Dose (mg/kg)	Ulcer index	Inhibition (%)
Disease control (distilled water)	—	4.0 ± 0.0	—
*Ranitidine*	100	3.5 ± 0.2	12.5%
	10	2.8 ± 0.3	29.3%

MCEO	50	2.5 ± 0.5^*∗*^	37.5%
	100	2.3 ± 0.6^*∗*^	41.8%

Data are expressed as mean ± SEM, *n* = 6, and ^*∗*^*p* < 0.05. Dunnett's test as compared to disease control value.

**Table 3:** Effect of MCEO on total lesion area (mm^2^) of gastric ulcer induced by indomethacin on rats.

Treatments	Dose (mg/kg)	Total lesion area (mm^2^)	Inhibition (%)
Disease control (distilled water)	—	36.7 ± 6.0	—
Ranitidine	100	6.2 ± 1.7^∗∗∗^	83.2
MCEO	10	20.5 ± 5.7^*∗*^	44.1
	50	12.5 ± 3.3^∗∗^	65.9
	100	7.3 ± 3.1^∗∗∗^	80.0

Data are expressed as mean ± SEM, *n* = 6, ^*∗*^*p* < 0.05, ^*∗∗*^*p* < 0.01, and ^*∗∗∗*^*p* < 0.005. Dunnett's test as compared to disease control value.

**Table 4:** Effect of MCEO on ulcer index of gastric ulcer induced by indomethacin on rats.

Treatments	Dose (mg/kg)	Ulcer index	Inhibition (%)
Disease control (distilled water)	—	3.3 ± 0.2	—
Ranitidine	100	2.0 ± 0.4^*∗*^	39.4
MCEO	10	2.7 ± 0.4	18.2
	50	2.3 ± 0.2	30.3
	100	1.8 ± 0.3^∗∗^	45.5

Data are expressed as mean ± SEM, *n* = 6, ^*∗*^*p* < 0.05 and ^∗∗^*p* < 0.01. Dunnett's test as compared to disease control value.

**Table 5:** Effect of MCEO on HCl/EtOH-induced oedema, epithelial disruption, and mucosa erosion in gastric tissues.

Groups	Grading system			
	Normal	Oedema and/or vacuolation	Epithelial disruption	Erosion extending to muscularised mucosae
Disease control (distilled water)	0.6 ± 0.2	3.7 ± 0.6	4.5 ± 0.7	3.3 ± 0.7
Ranitidine (100 mg/kg)	1.2 ± 0.2	1.7 ± 0.3^*∗*^	3.5 ± 0.5	1.3 ± 0.8
MCEO (10 mg/kg)	1.3 ± 0.3	2.3 ± 0.3	5.0 ± 0.6	2.0 ± 0.9
MCEO (50 mg/kg)	1.2 ± 0.3	3.0 ± 0.5	4.0 ± 0.6	1.3 ± 0.8
MCEO (100 mg/kg)	1.5 ± 0.2	2.6 ± 0.4	1.5 ± 0.7^∗∗^	0.7 ± 0.7

Data are expressed as mean ± SEM, *n* = 6, ^*∗*^*p* < 0.05, and ^∗∗^*p* < 0.01. Dunnett's test as compared with disease control value.

**Table 6:** Effect of MCEO on indomethacin-induced oedema, epithelial disruption, and mucosa erosion in gastric tissues.

Groups	Grading system			
	Normal	Oedema and/or vacuolation	Epithelial disruption	Erosion extending to muscularised mucosae
Disease control (distilled water)	0.8 ± 0.4	4.7 ± 0.8	6.5 ± 0.5	4.0 ± 1.0
Ranitidine (100 mg/kg)	2.7 ± 0.4^∗∗^	2.3 ± 0.3^∗∗^	3.5 ± 0.5^*∗*^	0.7 ± 0.7^*∗*^
MCEO (10 mg/kg)	2.3 ± 0.3^*∗*^	2.7 ± 0.4	4.5 ± 0.7	2.0 ± 0.9
MCEO (50 mg/kg)	2.2 ± 0.3^*∗*^	1.7 ± 0.3^∗∗^	3.0 ± 0.8^∗∗^	1.3 ± 0.8
MCEO (100 mg/kg)	2.5 ± 0.2^∗∗^	2.3 ± 0.8^*∗*^	3.0 ± 0.8^∗∗^	1.3 ± 0.8

Data are expressed as mean ± SEM, *n* = 6, ^*∗*^*p* < 0.05, and ^∗∗^*p* < 0.01. Dunnett's test as compared to disease control value.

**Table 7:** Effect of MCEO on gastric juice parameters in pylorus-ligation model in rats.

Groups	Gastric juice parameter				
	Ulcer area (mm^2^)	Protection (%)	Volume (ml)	pH (unit)	Total acidity (m equiv./L)
Disease control (distilled water)	12.3 ± 1.7	—	3.2 ± 0.4	2.2 ± 0.4	2927.0 ± 442.1
Ranitidine (100 mg/kg)	6.7 ± 0.6^*∗*^	46.0	2.2 ± 0.2	4.2 ± 0.2^∗∗∗^	2773.0 ± 452.7
MCEO (10 mg/kg)	8.3 ± 1.1	32.4	2.3 ± 0.4	2.9 ± 0.3	3020.0 ± 663.8
MCEO (50 mg/kg)	10.7 ± 1.5	13.5	4.1 ± 1.0	4.0 ± 0.2^∗∗∗^	5700.0 ± 59.1^∗∗^
MCEO (100 mg/kg)	7.0 ± 0.7^*∗*^	43.2	3.0 ± 0.8	4.0 ± 0.2^∗∗∗^	3380.0 ± 734.0

Data are expressed as mean ± SEM, *n* = 6, ^*∗*^*p* < 0.05, ^∗∗^*p* < 0.01, and ^∗∗∗^*p* < 0.005. Dunnett's test as compared to disease control value.

**Table 8:** Effect of MCEO on gastric wall mucus secretion in pylorus-ligation model in rats.

Treatments	Dose (mg/kg)	Gastric wall mucus (alcian blue mg/g wet tissue)
Disease control (distilled water)	—	1.1 ± 0.1
Ranitidine	100	1.9 ± 0.3^*∗*^
MCEO	10	1.6 ± 0.2
	50	2.1 ± 0.1^∗∗^
	100	2.1 ± 0.3^∗∗^

Data are expressed as mean ± SEM, *n* = 6, ^*∗*^*p* < 0.05, and ^∗∗^*p* < 0.01. Dunnett's test as compared to disease control value.

## Data Availability

The datasets supporting the conclusions of this study are included within the manuscript.
